# Retraction: Sensitive Visual Detection of AHPND Bacteria Using Loop-Mediated Isothermal Amplification Combined with DNA-Functionalized Gold Nanoparticles as Probes

**DOI:** 10.1371/journal.pone.0282453

**Published:** 2023-02-24

**Authors:** The PLOS ONE Editors

Following the publication of this article [1], concerns were raised regarding results presented in Figs 4, and 5. Specifically,

- The Fig 4B 17mM (1^st^ sample) and 3mM (3^rd^ sample) appear similar.
- The Fig 5A 10^4^ CFU/mL and 10^3^ CFU/mL appear similar.
- There appear to be multiple repetitive elements in the background of the last three lanes of the Fig. 5C results, and further repetitive elements in the underlying data initially provided for this figure.

The corresponding author clarified that in Fig 4B, the 17mM (1^st^ sample) result was replaced with the 3mM (3^rd^ sample) result. In addition, the corresponding author clarified that the Fig 5A 10^3^ CFU/mL result was inadvertently duplicated during figure preparation and that the color LAMP results were tested for dilutions ranging 10^6^−10^1^ only, as opposed to the 10^7^ CFU/mL reported in the results.

Following editorial questions regarding the originally provided underlying data, the corresponding author indicated that those data were incorrect and provided the original uncropped blot underlying the Fig 5C results, which confirmed that the image presented in Fig 5C was inappropriately manipulated, as the underlying data also demonstrated visible bands in the lanes marked as 10 CFU/mL and 1 CFU/mL. The original uncropped blot underlying the Fig 5C results calls into question the reported results that the LAMP-AuNP method showed similar sensitivity to LAMP followed by AGE.

In light of the concerns affecting multiple figure panels that question the integrity of these data, and the underlying data that call into question key results reported in this study, the *PLOS ONE* Editors retract this article.

NA and WK agreed with the retraction and apologise for the issues with the published article. JK, SS, PS, PP, and RS either did not respond directly or could not be reached.

## References

[1] Arunrut N, Kampeera J, Sirithammajak S, Sanguanrut P, Proespraiwong P, Suebsing R, et al. (2016) Sensitive Visual Detection of AHPND Bacteria Using Loop-Mediated Isothermal Amplification Combined with DNA-Functionalized Gold Nanoparticles as Probes. PLoS ONE 11(3): e0151769. 10.1371/journal.pone.0151769 27003504 PMC4803327

